# Heterogeneous evolutionary rates of *Pi2/9* homologs in rice

**DOI:** 10.1186/1471-2156-13-73

**Published:** 2012-08-19

**Authors:** Kejing Wu, Ting Xu, Changjiang Guo, Xiaohui Zhang, Sihai Yang

**Affiliations:** 1 State Key Laboratory of Pharmaceutical Biotechnology, School of Life Sciences, Nanjing University, Nanjing, 210093, China

**Keywords:** *Pi2/9*, Type I and Type II *R*-genes, Nucleotide diversity, Copy number variation, Positive selection, Gene conversion

## Abstract

**Background:**

The *Pi2/9* locus contains multiple nucleotide binding site–leucine-rich repeat (NBS-LRR) genes in the rice genome. Although three functional *R*-genes have been cloned from this locus, little is known about the origin and evolutionary history of these genes. Herein, an extensive genome-wide survey of *Pi2/9* homologs in rice, sorghum, *Brachypodium* and *Arabidopsis*, was conducted to explore this theme.

**Results:**

In our study, 1, 1, 5 and 156 *Pi2/9* homologs were detected in *Arabidopsis*, *Brachypodium*, sorghum and rice genomes, respectively. Two distinct evolutionary patterns of *Pi2/9* homologs, Type I and Type II, were observed in rice lines. Type I *Pi2/9* homologs showed evidence of rapid gene diversification, including substantial copy number variations, obscured orthologous relationships, high levels of nucleotide diversity or/and divergence, frequent sequence exchanges and strong positive selection, whereas Type II *Pi2/9* homologs exhibited a fairly slow evolutionary rate. Interestingly, the three cloned *R*-genes from the *Pi2/9* locus all belonged to the Type I genes.

**Conclusions:**

Our data show that the *Pi2/9* locus had an ancient origin predating the common ancestor of gramineous species. The existence of two types of *Pi2/9* homologs suggest that diversifying evolution should be an important strategy of rice to cope with different types of pathogens. The relationship of cloned *Pi2/9* genes and Type I genes also suggests that rapid gene diversification might facilitate rice to adapt quickly to the changing spectrum of the fungal pathogen *M. grisea*. Based on these criteria, other potential candidate genes that might confer novel resistance specificities to rice blast could be predicted.

## Background

Plants have evolved various mechanisms to protect themselves from pathogen invasion and colonization [1-3]. Firstly, plants use pattern-recognition receptors (PRRs) to recognize conserved pathogen-associated molecular patterns (PAMPs) which leads to a PAMP-triggered immunity (PTI)[2]. This innate immune system can be overcome by specialized microbial pathogens by secreting some small molecules (known as effectors). Therefore, plants have developed a second innate immune system to defend themselves. For example, plants employ surveillance proteins, encoded by *R*-genes to directly or indirectly monitor the presence of pathogen effector proteins, resulting in effector triggered immunity (ETI)[2,4]. Among these *R*-genes, the nucleotide-binding site–leucine-rich repeat (NBS-LRR) genes comprise the largest class and account for more than half of the known plant *R*-genes [3,5-8].

Previous studies have shown that most of the NBS-LRR genes are organized as tight complex clusters consisting of multiple copies [5,7-10]. Many studies indicated that such clustered arrangement contributed to the evolution of novel resistance specificities via gene conversion, recombination, or unequal crossing over [11,12]. As expected, extreme divergence, including a high level of polymorphisms, diversifying selections and sequence exchanges, have also been detected among these genes [12]. On the other hand, some NBS-LRR gene homologs locating at the same locus exhibited a different evolutionary pattern [13,14]. For example, two distinct categories of *RGC2* homologs, Type I and Type II, have been identified in *Lactuca*[14]. Type I genes consist of a large variety of *RGC2* homologs through mass sequence exchange events, and are generally diverse with no obvious allelic/orthologous relationships in different genotypes or their close relatives. In contrast, Type II homologs of *RGC2* evolve slowly and are highly conserved among accessions with rare sequence exchanges [14].

Rice blast, caused by the filamentous ascomycete *Magnaporthe grisea*, is one of the most devastating diseases that can seriously threaten to the global food supply [15]. Up to the present, 14 rice blast *R*-genes have been cloned [16,17]. The *Pi2/9* locus contains at least six known resistance genes specific to the fungal pathogen *M. grisea*, and three *R*-genes from this locus (*Pi2*, *Pi9*, and *Piz-t*) have been cloned [18,19]. Although all of these genes conferred broad spectrum resistance to rice blast, the resistance specificities were found to be different from one another. Genomic analysis of this locus in four wild rice species has shown that the copy number of *Pi2/9* homologs varies from 2 to 12 per genome, suggesting a complex evolutionary history for this *R*-gene locus that involved processes such as gene duplication and unequal crossing over, etc. [20]. However, little is known about copy number variation and the evolutionary patterns present within the *Pi2/9* locus. In order to gain a further understanding of the origin and evolutionary history of this locus, which could guide the discovery of these genetic variations, genomic sequences of *Pi2/9* homologs from 14 rice cultivars and 12 wild rice lines were collected for evolutionary analyses. Our data showed that, similar to *RGC2* homologs, two distinct types of *Pi2/9* homologs, Type I and Type II, were identified in rice lines at this locus. Although our findings suggest that this locus was involved in adaptation, it is important to consider that the different evolutionary patterns of *Pi2/9* homologs at a single locus reflect a complex evolutionary history of these genes.

## Results

### Identification and phylogenetic analyses of *Pi2/9* homologs

In order to study their evolutionary history, *Pi2/9* homologs were identified in 14 rice cultivars, 12 wild rice lines, and a single accession each of sorghum, *Brachypodium* and *A. thaliana*. Among these rice lines, the entire *Pi2/9* locus can be identified in 14 rice lines, including eight whole-genome and six BAC clone sequenced lines (Table 1). This locus could not be entirely reconstructed in the other 12 rice lines due to the partially sequenced fragments in this region only based on PCR productions or BAC-end sequences (Table 1; four rice varieties used for amplification with specific primers and eight wild rice lines from BAC end sequences libraries). A total of 156 *Pi2/9* homologs or fragments, including 73 entire NBS-LRRs and 83 NBS and/or LRR fragments, were detected in the total 26 rice lines (Table 1). Among the 14 rice lines with entire *Pi2/9* locus, the copy numbers of *Pi2/9*-like NBS-LRRs in each genome varied from two to ten, suggesting that these genes may have undergone rapid copy number evolution. In sorghum and *Brachypodium* genomes, 5 and 1 *Pi2/9* homologs were detected, respectively.

**Table 1:** **Number of *Pi2/9 *homologs in the rice, sorghum and *Brachypodium *genomes **

**Accession name**	**Species**	**Subspecies/Genome type**	**NBS-LRR*s***	***NBS or LRR*fragments**	**Total**	**Source of sequences**	**References**
Nippobare	*O. sativa*	*Japonica/AA*	6	1	7	WGS^b^	[41]
NK58	*O. sativa*	*Japonica/AA*	6	1	7	WGS	[46]
75-1-127	*O. sativa*	*Indica/AA*	8	1	9	BAC sequencing^c^	[18]
C101A51	*O. sativa*	*Indica/AA*	8	1	9	BAC sequencing	[19]
93-11	*O. sativa*	*Indica/AA*	4	0	4	WGS	[42]
PA64	*O. sativa*	*Indica/AA*	5	0	5	WGS	[43]
GLA4	*O. sativa*	*Indica/AA*	3	2	5	WGS	[46]
IR24	*O. sativa*	*Indica/AA*	4	1	5	WGS	This study
SH527	*O. sativa*	*Indica/AA*	4	1	5	WGS	This study
MH 63	*O. sativa*	*Indica/AA*	4	1	5	WGS	This study
GQ280269	*O. officinalis*	CC	6	0	6	BAC sequencing	[20]
GQ280268	*O. minuta*^*a*^	CC	7	3	10	BAC sequencing	[20]
GQ280267	*O. minuta*^*a*^	BB	2	0	2	BAC sequencing	[20]
GQ280266	*O. punctata*	BB	4	0	4	BAC sequencing	[20]
GQ280265	*O. nivara*	AA	2	0	2	BAC sequencing	[20]
IA96717	*O. glaberrimab*	AA	0	5	5	BES^d^	[47]
IA105491	*O. rufipogon*	AA	0	12	12	BES	[47]
IA105143	*O. alta*	CCDD	0	3	3	BES	[47]
IA100882	*O. australiensis*	EE	0	6	6	BES	[47]
IA101232	*O. brachyantha*	EE	0	2	2	BES	[47]
IA104502	*O. coarctatad*	HHKK	0	3	3	BES	[47]
IA102118	*O. granulata*	GG	0	6	6	BES	[47]
IA100821	*O. ridleyi*	HHJJ	0	6	6	BES	[47]
Q2436	*O. sativa*	*Indica/AA*	0	7	7	PCR	This study
GM2	*O. sativa*	*Indica/AA*	0	8	8	PCR	This study
Tetep	*O. sativa*	*Indica/AA*	0	6	6	PCR	This study
Tadukan	*O. sativa*	*Indica/AA*	0	7	7	PCR	This study
**Total**			73	83	156		
BTx623	*S. bicolor*		5	0	5	WGS	[49]
Bd21	*B.distachyon*		1	0	1	WGS	[50]

^*a*^*O. minuta* is an allotetroploid species with the BBCC genome constitution.

^b^ WGS, whole-genome sequencing.

^c^ BAC, bacterial artificial chromosome.

^d^ BES: BAC end sequence.

**Figure 1:**
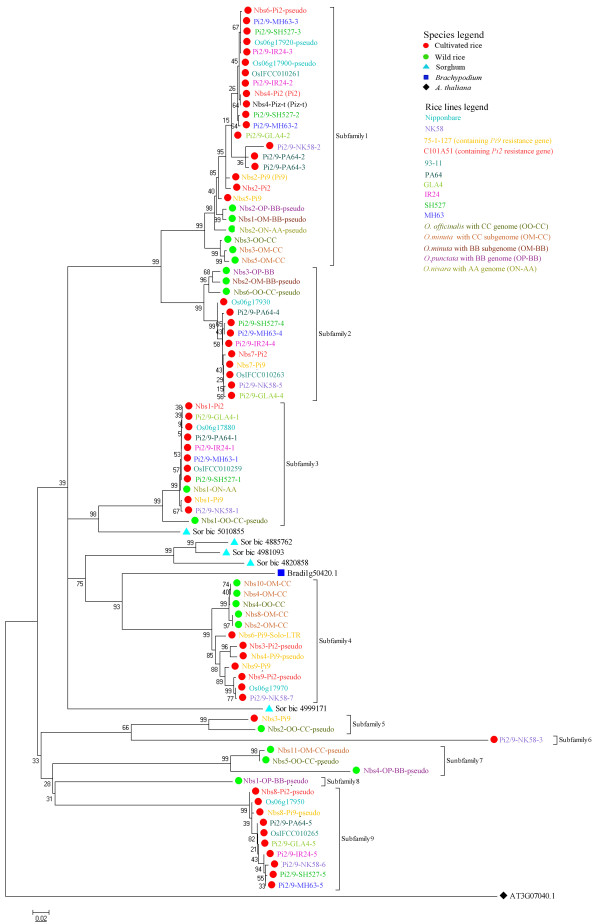
**Phylogenetic tree derived from *Pi2/9 *homologs in *Brachypodium, *sorghum and 14 rice lines. ** The *Arabidopsis* gene At3g07040.1 was used as an out group. *Pi2/9* homologs from the same rice line were indicated with the same color. The sequences of the *Pi2/9* loci from two cultivars (75-1-127, C101A51) and four wild rice species (*O. nivar*, *O. punctata*, *O. minuta*, and *O. officinalis*) were designated based on Qu et al., Zhou et al. and Dai et al. [18-20]. *Pi2/9* gene family members from lines, PA64, NK58, MH63, SH527, GLA4 and IR24, were designated *Pi2/9* followed by two suffixes separated by a hyphen. The first suffix identified the abbreviation of the cultivar or species designation, while the other numeric suffix represented the member’s order within the cluster. For the NBS-LRR genes in the wild rice species, additional suffixes denoting the sub genome designation were specified.

Using the *Pi2/9* homolog (AT3G07040.1) from *A. thaliana* as an out group, a phylogenetic tree was constructed based on the NBS domain of the NBS-LRR genes from sorghum, *Brachypodium* genomes and 14 rice lines with entire *Pi2/9* locus using neighbor-joining (NJ) method with the Kimura 2-parameter model (Figure 1). All rice sequences in the phylogenetic tree could be divided into seven multi-gene subfamilies and two single-gene subfamilies (Subfamily 6 and Subfamily 8) according to the topology (Figure 1). The multi-gene subfamilies were defined with high confidence bootstrap values (>90%) and high nucleotide similarity (>85%) among their members within the clade. To further confirm their topological relationships, another NJ tree was constructed by including some additional NBS-LRR genes from partially sequenced *Pi2/9* locus by PCR amplification or BAC-end sequence based homolog searches (see Additional file 1: Figure S1)*.* The supplemental tree displayed a similar topology to that of the original tree (Figure 1). All multi-gene clades were supported by high confidence bootstrap values and members of the same clade exhibited >85% nucleotide identity, consistent with the previously established phylogenetic tree. Interestingly, all of the NBS-LRR genes from sorghum and *Brachypodium* genomes were clustered near the clades of Subfamilies 3 and 4. To further investigate the origin and evolutionary history of these members in gramineous species, a collinear analysis of these genes was performed together with their flanking genes. Interestingly, at least one syntenic region pair can be detected between sorghum and rice genome. In this syntenic pair, one and seven *Pi2/9* homologs were found in the sorghum (*Sorbic_5010855*) and rice Nippobare genomes, respectively (see Additional file 2: Figure S2), suggesting that *Pi2/9* homologs had an ancient origin, which might predate the common ancestor of gramineous species. However, for the other four *Pi2/9* homologs in sorghum and one copy in *Brachypodium* genome, we did not find their corresponding syntenic pairs in the other grass species, suggesting that these *Pi2/9* homologs might have translocated to their present locations after these grass species split.

**Figure 2:**
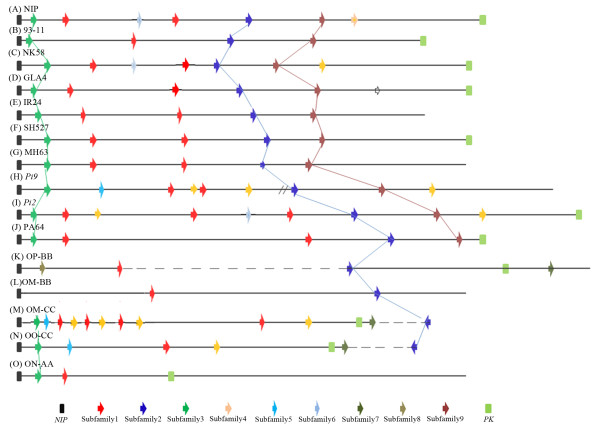
**Genome organization of the *Pi2/9 *locus in 14 rice lines with entire *Pi2/9 *locus.** The *Pi2/9* gene family members were indicated by colored arrows showing the transcriptional direction. *Pi2/9* homologs within the same subfamily were indicated with the same color. *Pi2/9*-GLA-6, not included in any clade (due to a partial sequence), was shown with an open *arrow*. The non-*Pi2/9* homologs, *NIP* (nitrate-induced protein gene) and *PK* (protein kinase gene) were indicated with rectangles. The gaps of undetermined length and very long distances between members were indicated by a double slash and dashed lines (not in scale), respectively. *Pi2/9* locus in (**A**) Nippobare, (**B**) 93–11, (**C**) NK58, (**D**) GLA4, (**E**) IR24, (**F**) SH527, (**G**) MH63, (**H**) 75-1-127 containing the *Pi9* gene, (**I**) C101A51 containing the *Pi2* gene, (**J**) PA64, (**K**) *O. punctata* with the BB genome constitution, (**L**) BB sub genome of *O. minuta*, (**M**) CC sub genome of *O. minuta*, (**N**) *O. officinalis* with the CC genome constitution,(**O**) *O. nivara* with the AA genome constitution.

On the other hand, copy number variations (CNVs) and genomic organization of the entire *Pi2/9* locus were further investigated in the 14 rice lines (Figure 2 and Table 2). The CNVs of *Pi2/9* homologs were also found in some subfamilies with obscured orthologous relationships in different rice lines, e.g. Subfamilies 1 and 4 (Figure 2 and Table 2). In contrast, some subfamilies, (e.g. Subfamilies 2, 3 and 9), had a relatively stable gene number with obvious orthologous relationships. In these families, homologs shared high levels of nucleotide similarity and conserved positions in their respective genomes (Figure 2 and Table 2). The distribution of sequences throughout another NJ tree, with some additional NBS-LRR genes from partially sequenced *Pi2/9* locus in Figure 1, further confirmed two different evolutionary patterns in rice (see Additional file 3: Table S1).

**Table 2:** **The distribution of *Pi2/9 *homologs in 14 rice lines with entire sequenced *Pi2/9 *locus **

**Species**	**Subfamily1**	**Subfamily2**	**Subfamily3**	**Subfamily4**	**Subfamily5**	**Subfamily6**	**Subfamily7**	**Subfamily8**	**Subfamily9**
75-1-127	2	1	1	3	1	0	0	0	1
C101A51	3	1	1	2	0	0	0	0	1
Nip	2	1	1	1	0	0	0	0	1
PA64	2	1	1	0	0	0	0	0	1
GLA4	1	1	1	0	0	0	0	0	1
NK58	1	1	1	1	0	1	0	0	1
IR24	2	1	1	0	0	0	0	0	1
SH527	2	1	1	0	0	0	0	0	1
MH63	2	1	1	0	0	0	0	0	1
93-11	1	1	1	0	0	0	0	0	1
OO-CC^a^	1	1	1	1	1	0	1	0	0
OM-CC^b^	2	0	0	4	0	0	1	0	0
OM -BB^c^	1	1	0	0	0	0	0	0	0
OP -BB^d^	1	1	0	0	0	0	1	1	0
ON-AA^e^	1	0	1	0	0	0	0	0	0
Average	1.60 ± 0.61	0.87 ± 0.34	0.80 ± 0.40	0.80 ± 1.22	0.13 ± 0.34	0.07 ± 0.25	0.20 ± 0.40	0.07 ± 0.25	0.67 ± 0.47

Subfamilies 1–9 referred to the corresponding phylogenetic tree in Figure 1, which contained 14 rice lines with entire *Pi2/9* locus.

^a^*Pi2/9* locus in *O. officinalis* with the CC genome constitution.

^b^*Pi2/9* locus in the CC subgenome of *O. minuta*.

^c^*Pi2/9* locus in the BB subgenome of *O. minuta*.

^d^*Pi2/9* locus in *O. punctata* with the BB genome constitution.

^e^*Pi2/9* locus in *O. nivara* with the AA genome constitution.

### Heterogeneous evolutionary rates of *Pi2/9* homologs within the same cluster

Previous studies have shown that NBS-LRR genes within a closely related group may evolve with a similar pattern, e.g. similar genome organization or *Ka/Ks* values [21]. As mentioned above for the rice lines, two types of *Pi2/9*-like genes were distinguished based on the CNVs in their subfamilies. To further investigate their evolutionary patterns, nucleotide diversities for the *Pi2/9* homologs in each subfamily were calculated. Because few members (*<*3) were found in Subfamilies 5, 6 and 8 (Table 2), these three subfamilies were excluded from further study. Due to the obscured orthologous relationships of gene copies in Subfamilies 1 and 4 between different rice lines, as expected, *Pi2/9* homologs in these two subfamilies had higher nucleotide diversity or divergence compared with *Pi2/9* homologs in the other three subfamilies (Table 3). Even when the nucleotide diversities were calculated between the least-divergent pairs of any two cultivars, Subfamilies 1 and 4 still had higher diversities than did in Subfamilies 2 and 3 (Table 3).

**Table 3:** Nucleotide diversity and positive selection within subfamilies

**Subfamily**	**Gene No.**	**π**			**θ**		***Ka/Ks*(LRR)**^**d**^	**REL**	
		**W**^**a**^	**C**^**b**^	**C**_**min**_^**c**^	**W**	**C**		**Break points**	**Positively selected sites**
1	24	0.0600	0.0542	0.0378 ± 0.0257	0.0585	0.0912	1.28	4	17
2	13	0.0292	0.0035	0.0044 ± 0.0032	0.0295	0.0052	0.06	1	1
3	12	0.0545	0.0029	0.0029 ± 0.0031	0.0545	0.0033	0.39	1	1
4	12	0.0308	0.0436	0.0079 ± 0.0060	0.0276	0.0467	1.41	4	12
7	3	0.0823	-	-	0.1166	-	1.26	5	28
9	9	-	0.0247	0.0247 ± 0.0412	-	0.0066	0.47	5	0

^a^ C, rice cultivars.

^b^ W, wild rice.

^c^ Nucleotide diversity between the least-divergent pairwise of any two cultivars.

^d^ The ratios of non-synonymous (*Ka*) to synonymous (*Ks*) substitutions of the × ×L × L × × motif.

To explore whether different selective constrains exist on the two types of *Pi2/9* homologs, the ratio of non-synonymous to synonymous amino acid substitution (*Ka/Ks*) in the LRR core regions (× × L × L × × motifs; L = Leu or other aliphatic amino acid; × = any amino acid) was calculated. According to the hypothesis that synonymous changes approximate the neutral rate of molecular evolution, *Ka > Ks* provides solid evidence of positive selection for amino acid substitution [22]. On the other hand, *Ka < Ks* is suggestive of purifying selection. Comparison of LRR core regions within different subfamilies can provide an appropriate method to evaluate the range of *Ka/Ks* and the strength of selection during the evolutionary history of the two types of *Pi2/9* homologs [22,23]. Table 3 shows that significant *Ka > Ks* was detected on core regions between homologs within Subfamilies 1 and 4. However, in Subfamilies 2, 3 and 9, purifying selection was observed (Table 3). The results were further supported by the positively selected sites detected by the HyPhy package using the REL method. In the Type I *Pi2/9* homologs (Subfamilies 1 and 4), 29 positively selected sites were found. The number was significant higher than that in the Type II subfamilies (2, 3 and 9; Table 3), where only two were present.

Notably, Subfamily 7 only containing wild rice genes had exceptionally high levels of polymorphism (0.0823; Table 3). In addition, strongly positive selection was detected in the core region of the LRR in this subfamily (*Ka/Ks* =1.26; Table 3).

### Detection of gene conversion events

**Figure 3:**
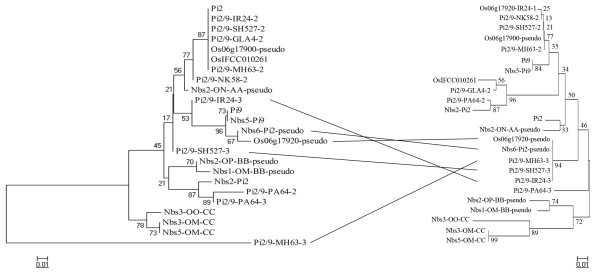
Distance trees constructed for LRR4 (left) and LRR13 (right) of Subfamily 1 sequences.

Gene conversion is a process in which one segment of a DNA sequence is copied onto another segment of DNA, and is considered to be an important evolutionary force in the evolution of multigene families. To further investigate whether gene conversion contributed to nucleotide diversity differently in the two types of *Pi2/9* gene families, we used the software GENECONV to detect possible gene conversion events. A total of 77 independent sequence exchange events were predicted in all fragments using GENECONV (*P* < 0.05). Among these events, 66 occurred within Type I *Pi2/9* homologs, whereas just 11 occurred between Type I and Type II homologs and no gene conversion event was identified within the Type II genes. The same analysis within each subfamily also indicated that more sequence exchange existed in Type I (Subfamilies 1 and 4) than in Type II groups (Subfamilies 2, 3 and 9). Distance trees were constructed for each of the 17 LRR units to investigate sequence exchange between LRR units among Subfamily 1 *Pi2/9* homologs (Type I genes). Interestingly, the tree topologies of these 17 LRR units were extremely different from one another. Our data show that there were two main differences. One was that the different LRR units from the same gene did not show correlation between the LRR trees, the other was that genes with high sequence similarity at one LRR were not always similar at another (Figure 3). For example, when comparing the trees for LRR4 and LRR13 unit, five identical sequences were clustered within a single clade in the LRR13 tree (Os06g17920, Nbs6-*Pi2*, *Pi2/9*-MH63-3, *Pi2/9*-SH527-3 and *Pi2/9*-IR24-3), while the sequences from these five *Pi2/9* homologs were distributed throughout the LRR4 tree (Figure 3). These mosaic distributions of LRRs within a single gene suggest that frequent sequence exchanges also may occur between LRRs within the same gene.

### Genetic structure of the *Pi2/9* homologs between cultivated and wild rice

Based on the phylogenetic tree of *Pi2/9* gene homolog sequences, the cultivated and wild rice lines were clearly separated into different clades in most subfamilies (Figure 1). In order to clarify the phylogenetic relationships and genetic structure of *Pi2/9* homologs between species, divergences (*D*_*xy*_) were calculated. In addition, *F*_*st*_ and *S*_*nn*_ were used to explore the genetic differentiation (Table 4). As expected, a high level of *D*_*xy*_ was observed between rice cultivars and their wild relatives, especially in Type I genes (7.90% in Subfamily 1; 7.61% in Subfamily 4). In addition, except for Subfamily 3, significant genetic differentiation between cultivated and wild rice was detected based on *F*_*st*_ and *S*_*nn*_. In contrast, significant genetic differentiation was not observed between the *indica* and *japonica* subspecies in these subfamilies (Table 4).

**Table 4:** **Analysis of genetic structure between cultivated and wild rice or *indica *and *japonica ***

**Subfamily**	**Dxy**	**Genetic differentiation between W and C**		**Genetic differentiation between Ind and Jap**	
		***Snn***	***Fst***	***Snn***	***Fst***
1	0.0790	0.96***	0.16*	0.14	0.04
2	0.0430	1.00**	0.81***	0.60	0.40
3	0.0245	0.83	0.31	1.00	0.06
4	0.0761	0.01**	0.37**	0.21	0.01

*, 0.01 < *P* <0.05; **, 0.001 < *P* <0.01; ***, *P* <0.001.

Previous research has demonstrated that *O. sativa* originated from common wild rice and that there was significantly more genetic variation present in wild rice as compared to cultivars[24]. As shown in Table 3, the values of π for Subfamilies 1 and 4, which represented Type I genes, were 0.0542 and 0.0436 in rice cultivars, respectively, while wild rice relatives in the same family had nucleotide diversities of 0.0600 and 0.0308, respectively. The nucleotide diversities (π) in cultivated rice are as much as or even greater than that in wild rice. To further investigate the genetic variation among populations, the average nucleotide diversity (θ), which was less affected by the frequency of nucleotide substitutions, was analyzed [25]. As expected, the results of this analysis also show that there was an excess of nucleotide polymorphism maintained in rice cultivars in the Type I sequence group.

In addition, previous studies have demonstrated that the *Pi9* gene was introgressed from *O. minuta* into the isogenic line 75-1-127[18]. Our analyses, however, showed that the *Pi9* gene embedded in Subfamily 1 with its homologs from the cultivated lines were different from other homologs in *O. minuta,* suggesting that *Pi9* might not originate from *O. minuta*, but rather from cultivars by gene conversion during the process of artificial selection*.*

## Discussion

### Different evolutionary rates of *Pi2/9* homologs

In the last decade, many *R*-genes have been cloned and sequenced from a variety of diverse plant species [9,26]. However, only a few of these loci have been analyzed in any detail. For example, heterogeneous evolutionary rates within the same *R*-locus, defined as Type I and Type II *R*-genes, were detected in *RGC2* genes in lettuce and *RPP8* genes in *Arabidopsis.*[13,27]. In these studies, Type II genes were shown to evolve more slowly than Type I genes. Classical genetic and molecular data show that plant resistance genes are frequently organized as clusters in genomes[5,7,8,10]. The *Pi2/9* locus belongs to a complex NBS-LRR gene cluster and contains at least six resistance alleles with specificities against the rice blast pathogen. Three of these genes, *Pi2*, *Pi9*, and *Piz-t,* which confer broad-spectrum resistance to *M. grisea*, have been cloned.

Our data showed that, similar to the RGC2 and RPP8 loci, two distinct types of homologs (Type I and Type II) were also found within the *Pi2/9* locus in different rice lines, and that the two types exhibited different evolutionary patterns. In Type I *Pi2/9* homologs, including members of Subfamilies 1 and 4, higher CNVs, higher nucleotide diversity or/and divergence, obscured orthologous relationships between different rice lines, frequent sequence exchanges between members and significant *Ka > Ks* ratios were observed compared with Type II homologs, suggesting higher evolutionary rates in Type I *Pi2/9* homologs. Interestingly, the three *R*-genes (*Pi2*, *Pi9*, and *Piz-t*) cloned from this locus were all clustered in Subfamily 1 (Type I genes), also suggesting that the rapid gene diversification of *Pi2/9* homologs may be a strategy for rice to adapt quickly to the changing spectrum of the fungal pathogen *M. grisea*.

Previous studies have shown that different types of resistance genes are found in some complex loci where multiple homologous genes are clustered together [11,23,28,29]. Within these complex *R*-gene loci, gene duplication and subsequent sequence diversification might play an important role in the rapid evolution of *R*-genes [30]. Although point mutations may have an impact on *Pi2/9* homolog variations, sequence exchanges, including sequence crossover, unequal crossing over and gene conversion, were much more important in the evolution of diverse *Pi2/9* homologs. Our analysis of sequence exchanges has shown that Type I *Pi2/9* homologs appear to have been subjected to frequent conversion events, whereas Type II genes are not, indicating an unequal evolutionary history between these two type genes. On the other hand, more non-synonymous amino acid substitutions than synonymous substitutions were detected in Type I *Pi2/9* homologs, suggesting that different selective pressures acted on these two types of *Pi2/9* homologs. These results are consistent with previous views that *R*-gene clusters might be reservoirs for rapid evolution of novel resistance specificities that can occur via frequent sequence exchange. The heterogeneous patterns of evolution of *Pi2/9* homologs within the same locus also might be the result of natural selection: the Type I genes that display rapid evolutionary rates may recognize the non-conserved pathogen molecules, whereas Type II genes most likely recognize more conserved pathogen effector molecules, and may confer some durable resistance based on their highly conserved sequences. In addition, the lower copy number of Type II genes could possibly avoid a fitness cost [31]. All these suggest that the diverse evolutionary patterns observed in *Pi2/9* homologs could be an important strategy for adaptation, which allows rice to cope with different types of pathogens.

### Potential resistance gene candidates of *Pi2/9* alleles in rice

Previous studies have shown that for NBS-LRR genes, frequent unequal crossing over to generate gene duplication and their subsequent sequence exchanges, either through gene conversion or recombination, can facilitate the accumulated non-synonymous substitutions more efficiently to create novel *R*-genes[12]. The exceptional diversity of *R*-genes is an important strategy for species to adapt to the quickly changing spectrum of pathogen specificities. For example, the *RPP13*, *RPP8*, *RPP5*, *RGC2* and *L* loci have extremely high levels of sequence polymorphism [27,32-37]. In our study, *Pi2/9* homologs in Type I families clearly maintained high levels of variation, indicating that pathogen-mediated selection pressures act on these resistance specificities to evolve new *R*-genes. In fact, dynamic copy number variation, frequent sequence exchanges and strong positive selection might be the major force driving the rapid evolution and diversification of *Pi2/9* homologs. Many studies have also confirmed that positive selection of the LRR domains allows for altered recognition specificities [36,38,39]. Since the rapid adaptive evolution of *R*-genes is apparently important to respond to the quickly changing spectrum of pathogens in the environment, the ratio of nucleotide substitutions that lead to amino acid replacements (*Ka*) to nucleotide substitutions that do not result in an amino acid change (*Ks*) in LRR regions might be a reasonable parameter for detecting the functional genes.

As expected, all three cloned *R*-genes from the *Pi2/9* locus, *Pi2, Pi9* and *Piz*-*t*, were clustered in Subfamily 1 with evidences for strong positive selection (*Ka/Ks* = 1.28) and frequent gene conversions, which have been involved in the specific recognition of different pathogen isolates. Similarly, rapid evolution was also observed in *Pi2/9* homologs in Subfamily 4 with high *Ka/Ks* (1.41) and frequent gene conversions, which additionally contained representative *Pi2/9* homologs from other two gramineous species, indicating that the *Pi2/9* homologs in this subfamily may also encode potential candidates for resistance genes to as yet unknown strains of *M. grisea*. In addition, another candidate subfamily for resistance genes might be Subfamily 7, which is a wild rice specific cluster that displays high *Ka/Ks* (1.26) and high level of polymorphisms, suggesting that some homologs within this subfamily may confer novel resistance specificities to rice blast.

According to the *Fst* and *Snn* statistic of *Pi2/9* homologs, we noted that the significant genetic differentiation was absent between indica and japonica subspecies, which might result from the selective pressure imposed by similar groups of pathogens. However, the significant genetic differentiation was observed between cultivars and wild rice species, suggesting that *Pi2/9* homologs have undergone artificial selection. The availability of wild germplasms could be an important tool to expand the gene pool of cultivars.

## Conclusions

Our data show that the *Pi2/9* locus have an ancient origin predating the common ancestor of gramineous species and the genes have undergone rapid copy number evolution in both wild and cultivated rice lines. The *Pi2/9* homologs have been classified into two categories based on their distinct evolutionary patterns (Type I and Type II). Common characteristics of slowly evolving *R-*genes (Type II) were: copy-number uniformity, obvious orthologous relationships and low nucleotide diversity. On the contrary, frequent copy number variations, high levels of nucleotide diversity, and obscured orthologous relationships were observed in Type I genes with fast evolutionary rate. The existence of two types of *Pi2/9* genes was further supported by selective constrains and the frequency of sequence exchange. Unlike Type II genes, Type I genes exhibited frequent sequence exchanges and strong positive selection. Furthermore, the three *R*-genes cloned from the *Pi2/9* locus were also clustered with Type I genes. All the results suggest that the rapid gene diversification of *Pi2/9* homologs may be a strategy for rice to adapt quickly to the changing spectrum of the fungal pathogen *M. grisea*. Therefore, some homologs within the same subfamilies of Type I genes might be candidates for resistance genes to different spectrum of pathogens.

## Methods

### Data sources

Eight fully sequenced rice (*Oryza sativa*) genomes were employed in this research. Nipponbare (*O. sativa* L*.* ssp. *japonica*, Release 6.1) whole-genome map-based sequences were obtained from the International Rice Genome Sequencing Project [40,41]. Two whole genomes assembled by shotgun sequencing, 93–11 and PA64s (*O. sativa* L*.* ssp. *indica*), were obtained from the Beijing Genomics Institute (BGI) database [42-44]. NK58 (*japonica*) and GLA4 (*indica*) whole-genome sequences obtained by sequencing-by-synthesis technology were downloaded from the National Center for Genome Resources [45,46]. The other three resequenced *indica* genomes, IR24, SH527 and MH63, obtained using high-throughput sequencing technology from Illumina, were kindly provided by Ping Li (Sichuan Agricultural University, China).

In addition, bacterial artificial chromosome (BAC) end sequences from 12 species libraries representing the 10 distinct genome types were downloaded [47,48]. Because previous studies have completely sequenced BAC clones spanning the *Pi2/9* locus in two cultivars, C101A51 and 75-1-127, which contained the *Pi2* and *Pi9* resistance genes, respectively, and four wild rice individuals representing *O. nivara*, *O. punctata*, *O. offcinalis*, and *O. minuta*, we just downloaded these sequences from online database [18-20]. Among them, *O. minuta* is an allotetroploid species with the BBCC genome constitution. To investigate the evolutionary history of *Pi2/9* homologs in gramineous species, the sorghum [49], and *Brachypodium* (*B. distachyon*, v2.0)[50] assembly and gene models were obtained from the Joint Genome Institute [51] and the *Brachypodium distachyon* database [52], respectively. *Arabidopsis thaliana* sequences used as out groups for phylogenetic analysis were downloaded from the *Arabidopsis* Information Resource (TAIR) [53].

### PCR amplification and DNA sequencing

Previous studies have shown that rice cultivars Q2436, Tadukan, GM2 and Tetep express a high level of resistant to rice blast [54,55]. Therefore, these four cultivars were chosen for investigation of gene homolog variation at the *Pi2/9* locus. Locus-specific primers were designed based on conserved sites adjacent to the borders of the NBS domain (see Additional file 3 Table S2). As *R*-genes are often organized as tandem arrays with varying copy numbers between populations, PCR products were cloned into a PGEM-T Easy Vector (Promega), and >20 colonies from each cultivar were then sequenced separately until no new homolog sequence could be identified. PCR products were sequenced on an ABI3100A automated sequencer. All DNA sequences have been submitted to the GenBank databases (accession numbers JX258293–JX258320).

### Identification of *Pi2/9* homologs

To identify *Pi2/9* homologs in genomic sequences, BLAST and hidden Markov model (HMM) search methods were employed [21,56]. Nucleotide sequences of *Pi9* and its paralogs from the *indica* rice line 75-1-127 [18] were used as queries in BLASTN searches against the genomes of cultivated rice lines (Nippobare, 93–11 and PA64s), sorghum, *Brachypodium*, and *A. thaliana*. In order to filter out most of the spurious hits, the threshold expectation value was set to 1E-100. The candidate sequences were further surveyed to determine whether they encoded NBS and LRR motifs using the Pfam database [57] and SMART protein motif analyses [58].

*Pi2/9* homologs in the resequenced rice genomes (NK58, GLA4, IR24, SH527 and MH63) were retrieved by mapping reads to the candidate *Pi2/9* homologs from Nippobare, 93–11 and PA64s genomes which were used as references [59]. Aligned reads were picked up with a minimum cut-off of 90% identity over a read. Only uniquely aligned reads (reads mapped to unique locations in these reference sequences) were retained and low-quality base sites (base-quality Q score in Phred scale <20) were removed. Similarly, BLASTN was also employed using the known *Pi2/9* homologs as the queries to find *Pi2/9* homologs in the BAC-end sequences.

### Sequence alignment and phylogenetic analyses

The amino acid sequences were first aligned with the program MUSCLE using default options [60], and MEGA v5.0[61] was used subsequently to manually correct the alignments. The resulting amino acid sequence alignments were then used to guide the alignments of the corresponding nucleotide coding sequences (CDSs). Based on the alignment results, phylogenetic trees were generated using the bootstrap neighbor-joining (NJ) method with the Kimura two-parameter model in MEGA v5.0. The stability of internal nodes was assessed by bootstrap analysis with 1,000 replicates.

To detect positive selection, the ratios of no synonymous to synonymous nucleotide substitutions (*Ka/Ks*) were calculated using DnaSP version v5.0 [62] on the full-length coding sequences (CDSs) and the xxLxLxx motifs of the LRR domain, which is regarded as the determinant of recognition specificity for Avr factors [63]. To further detect positively selective sites, we used the HyPhy package with the random effects likelihood (REL) method as implemented on the Data monkey web server [64-66].

Nucleotide diversity (π) was estimated with the Jukes and Cantor correction [67] and average nucleotide diversity (θ) from the number of polymorphic segregating (S) sites [25] using DnaSP v5.0. The parameter θ could better measure the richness of genetic variation among populations because this type of variation was less affected by the frequency of nucleotide substitutions. The divergences between species (*Dxy*) were obtained with the Jukes and Cantor correction. GENECONV1.81 was used to investigate sequence exchanges [68]. The default setting of 10,000 permutations was used for the analysis. The statistical significance of gene conversion events was defined as a global permutation *P* value of <0.05.

### Population genetic analyses

To assess genetic differentiation of subpopulations, two sequence-based statistical tests, *Fst* and *Snn*, were applied. It has been proposed that population specific *Fst* could measure the genetic variance between populations divided by the total genetic variance of the entire population [69]. The ARLEQUIN version 3.11 software package for population genetics data analysis [70] was used to compute genetic distances between cultivated and wild rice or *indica* and *japonica* subspecies. The statistical significance (*P* value) of pair wise *Fst* was determined by permuting the data 1,000 times. The nearest-neighbors statistic (*Snn*) is a measure of how often the nearest neighbors of sequences are found in the same locality. Previous studies demonstrated that *Snn* was the most powerful statistic under all conditions examined [71]. The statistical significance of pair wise *Snn* values was determined by permuting the data 1,000 times in DnaSP v5.0.

## Abbreviations

PRRs: Pattern-Recognition Receptors; PAMPs: Pathogen-Associated Molecular Patterns; PTI: PAMP-Triggered Immunity; ETI: Effector Triggered Immunity; NBS-LRR: Nucleotide-Binding Site–Leucine-Rich Repeat; CNVs: Copy Number Variations; BAC: Bacterial Artificial Chromosome; BES: BAC End Sequence.

## Competing interests

The authors declare that they have no competing interests.

## Authors' contributions

SY, XZ and KW designed the study. KW and XZ contributed extensively to the bioinformatic analyses. TX and CG performed PCR experiments. SY, TX and KW wrote the manuscript. SY, KW and XZ prepared and revised the manuscript. All authors read and approved the final manuscript.

## Supplementary Material

Additional file 1 **Figure S1.** Phylogenetic tree derived from NBS domains of homologs in completely and partially sequenced *Pi2/9* locus. To further confirm the topological relationships in Figure 1, another NJ tree was constructed by including some additional NBS-LRR genes from partially sequenced *Pi2/9* locus by PCR amplification or BAC-end sequence based homolog searches. (PDF 92 kb) Click here for file

Additional file 2 **Figure S2.** Gene collinearity in orthologous regions between rice and sorghum. One syntenic region pair was detected between rice and sorghum genome. Each gene was indicated as horizontal lines. Orthologous genes were joined by solid lines. The genes marked by red lines represented *Pi2/9* homologs. (PDF 32 kb) Click here for file

Additional file 3 **Table S1.** The distribution of *Pi2/9* homologs corresponding to Figure 1. The gene copy number variations of *Pi2/9* homologs were found in subfamilies. Subfamilies 1–10 referred to corresponding phylogenetic tree in Figure 1, which was derived from all *Pi2/9* homologs in completely and partially sequenced *Pi2/9* locus. **Table S2** as XLS Sequences of oligonucleotide primers used in PCR amplification. Locus-specific primers were designed based on conserved sites of the consensus NBS domain to investigate Pi2/9 homolog variation in four cultivars, Q2436, Tadukan, GM2 and Tetep. (XLS 30 kb) Click here for file
