# Supplementary material for: Heterogeneous evolutionary rates of Pi2/9 homologs in rice
Source: BMC Genet. 2012 Aug 19;13:73. doi: 10.1186/1471-2156-13-73 (PMC3492116; doi:10.1186/1471-2156-13-73)
Supplement: Additional file 1 — Figure S1. Phylogenetic tree derived from NBS domains of homologs in completely and partially sequenced Pi2/9 locus. To further confirm the topological relationships in Figure 1, another NJ tree was constructed by including some additional NBS-LRR genes from partially sequenced Pi2/9 locus by PCR amplification or BAC-end sequence based homolog searches. (PDF 92 kb) [file 1471-2156-13-73-S1.pdf]

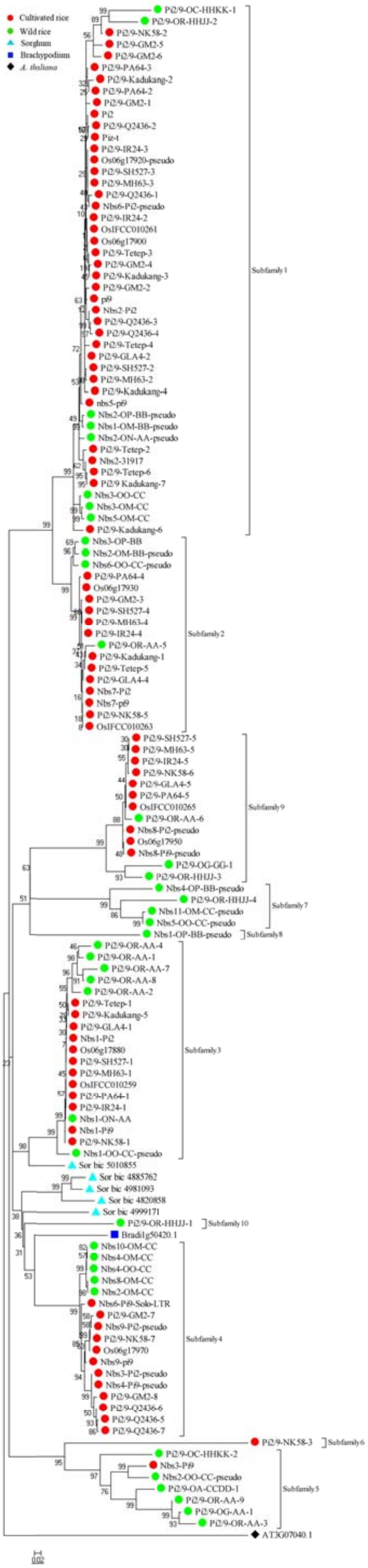

Cultivated rice
Wild rice
Sorghum
Brachypodium
A. thaliana
Pi2/9-OC-HHKK-1
Pi2/9-OR-HHJJ-2
Pi2/9-NK58-2
Pi2/9-GM2-5
Pi2/9-GM2-6
Pi2/9-PA64-3
Pi2/9-Kadukang-2
Pi2/9-PA64-2
Pi2/9-GM2-1
Pi2
Pi2/9-Q2436-2
Piz-t
Pi2/9-IR24-3
Os06g17920-pseudo
Pi2/9-SH527-3
Pi2/9-MH63-3
Pi2/9-Q2436-1
Nbs6-Pi2-pseudo
Pi2/9-IR24-2
OsIFCC010261
Os06g17900
Pi2/9-Tetep-3
Pi2/9-GM2-4
Pi2/9-Kadukang-3
Pi2/9-GM2-2
pi9
Nbs2-Pi2
Pi2/9-Q2436-3
Pi2/9-Q2436-4
Pi2/9-Tetep-4
Pi2/9-GLA4-2
Pi2/9-SH527-2
Pi2/9-MH63-2
Pi2/9-Kadukang-4
nbs5-pi9
Nbs2-OP-BB-pseudo
Nbs1-OM-BB-pseudo
Nbs2-ON-AA-pseudo
Pi2/9-Tetep-2
Nbs2-31917
Pi2/9-Tetep-6
Pi2/9 Kadukang-7
Nbs3-OO-CC
Nbs3-OM-CC
Nbs5-OM-CC
Pi2/9-Kadukang-6
Subfamily 1
Nbs3-OP-BB
Nbs2-OM-BB-pseudo
Nbs6-OO-CC-pseudo
Pi2/9-PA64-4
Os06g17930
Pi2/9-GM2-3
Pi2/9-SH527-4
Pi2/9-MH63-4
Pi2/9-IR24-4
Pi2/9-OR-AA-5
Pi2/9-Kadukang-1
Pi2/9-Tetep-5
Pi2/9-GLA4-4
Nbs7-Pi2
Nbs7-pi9
Pi2/9-NK58-5
OsIFCC010263
Subfamily 2
Pi2/9-SH527-5
Pi2/9-MH63-5
Pi2/9-IR24-5
Pi2/9-NK58-6
Pi2/9-GLA4-5
Pi2/9-PA64-5
OsIFCC010265
Pi2/9-OR-AA-6
Nbs8-Pi2-pseudo
Os06g17950
Nbs8-Pi9-pseudo
Pi2/9-OG-GG-1
Pi2/9-OR-HHJJ-3
Subfamily 9
Nbs4-OP-BB-pseudo
Pi2/9-OR-HHJJ-4
Nbs11-OM-CC-pseudo
Nbs5-OO-CC-pseudo
Subfamily 7
Nbs1-OP-BB-pseudo
Subfamily 8
Pi2/9-OR-AA-4
Pi2/9-OR-AA-1
Pi2/9-OR-AA-7
Pi2/9-OR-AA-8
Pi2/9-OR-AA-2
Pi2/9-Tetep-1
Pi2/9-Kadukang-5
Pi2/9-GLA4-1
Nbs1-Pi2
Os06g17880
Pi2/9-SH527-1
Pi2/9-MH63-1
OsIFCC010259
Pi2/9-PA64-1
Pi2/9-IR24-1
Nbs1-ON-AA
Nbs1-Pi9
Pi2/9-NK58-1
Nbs1-OO-CC-pseudo
Subfamily 3
Sor bic 5010855
Sor bic 4885762
Sor bic 4981093
Sor bic 4820858
Sor bic 4999171
Pi2/9-OR-HHJJ-1
Subfamily 10
Bradi1g50420.1
Nbs10-OM-CC
Nbs4-OM-CC
Nbs4-OO-CC
Nbs8-OM-CC
Nbs2-OM-CC
Nbs6-Pi9-Solo-LTR
Pi2/9-GM2-7
Nbs9-Pi2-pseudo
Pi2/9-NK58-7
Os06g17970
Nbs9-pi9
Nbs3-Pi2-pseudo
Nbs4-Pi9-pseudo
Pi2/9-GM2-8
Pi2/9-Q2436-6
Pi2/9-Q2436-5
Pi2/9-Q2436-7
Subfamily 4
Pi2/9-NK58-3
Subfamily 6
Pi2/9-OC-HHKK-2
Nbs3-Pi9
Nbs2-OO-CC-pseudo
Pi2/9-OA-CCDD-1
Pi2/9-OR-AA-9
Pi2/9-OG-AA-1
Pi2/9-OR-AA-3
Subfamily 5
AT3G07040.1
0.02

Figure S1 Phylogenetic tree derived from NBS domains of homologs in completely and partially sequenced *Pi2/9* locus.
