# Supplementary material for: Heterogeneous evolutionary rates of Pi2/9 homologs in rice
Source: BMC Genet. 2012 Aug 19;13:73. doi: 10.1186/1471-2156-13-73 (PMC3492116; doi:10.1186/1471-2156-13-73)
Supplement: Additional file 2 — Figure S2. Gene collinearity in orthologous regions between rice and sorghum. One syntenic region pair was detected between rice and sorghum genome. Each gene was indicated as horizontal lines. Orthologous genes were joined by solid lines. The genes marked by red lines represented Pi2/9 homologs. (PDF 32 kb) [file 1471-2156-13-73-S2.pdf]

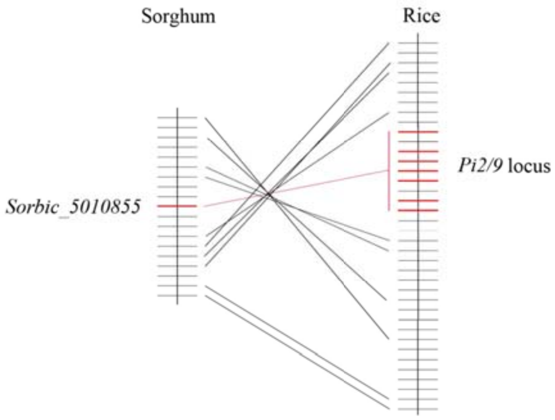

Figure S2 Gene collinearity in orthologous regions between rice and sorghum.

Each gene was indicated as horizontal lines. Orthologous genes were joined by solid lines. The genes marked by red lines represented *Pi2/9* homologs.
